# Selection of lactic acid bacteria for biopreservation of salmon products applying processing-dependent growth kinetic parameters and antimicrobial mechanisms

**DOI:** 10.1016/j.heliyon.2023.e19887

**Published:** 2023-09-09

**Authors:** Jelena Stupar, Sunniva Hoel, Sigrid Strømseth, Jørgen Lerfall, Turid Rustad, Anita Nordeng Jakobsen

**Affiliations:** Norwegian University of Science and Technology, Department of Biotechnology and Food Science, NO-7491, Trondheim, Norway

**Keywords:** Lactic acid bacteria (LAB), Biopreservation, Fresh salmon, Growth prediction, Process parameters

## Abstract

Biopreservation using lactic acid bacteria (LAB) is a promising technology to prevent the growth of pathogenic microorganisms in fresh and mildly processed food. The main aim of this study was to select LAB, originally isolated from ready-to-eat (RTE) seafood, for biopreservation of fresh salmon and processed salmon products. Ten LAB strains (five *Carnobacterium* and five *Leuconostoc*) were selected based on previously demonstrated bioprotective properties to investigate their antimicrobial mechanisms and temperature-dependent growth kinetics in a sterile salmon juice model system. Furthermore, five strains (three *Carnobacterium* and two *Leuconostoc*) were selected to test process-dependent growth kinetic parameters relevant to the secondary processing of salmon. Two strains (*Carnobacterium maltaromaticum* 35 and *C. divergens* 468) showed bacteriocin-like activity against *Listeria innocua*, while inhibitory effect of cell-free supernatants (CFS) was not observed against *Escherichia coli*. All selected strains were able to grow in sterile salmon juice at tested temperatures (4, 8, 12 and 16 °C), with specific growth rates (*μ*) ranging from 0.01 to 0.04/h at 4 °C and reaching a maximum population density of 8.4–9 log CFU/ml. All five strains tested for process-dependent growth kinetic parameters were able to grow in the range of 0.5–5% NaCl and 0.13–0.26% purified condensed smoke (VTABB and JJT01), with inter- and intraspecies variation in growth kinetics. According to the temperature-dependent growth kinetics and antimicrobial assay results, two strains, *Leuconostoc mesenteroides* 68 (Le.m.68) and *C*. *divergens* 468 (C d.468), were selected for *in situ* test to validate their ability to grow in vacuum-packed fresh salmon at 4 °C. Both strains were able to grow at maximum growth rates of 0.29 ± 0.04/d for Le. m.68 and 0.39 ± 0.06/d for C.d.468, and their final concentrations were 7.91 ± 0.31 and 8.02 ± 0.25 log CFU/g, respectively. This study shows that LAB, originally isolated from RTE seafood, have promising potential as bioprotective strains in fresh and processed salmon products.

## 1 Introduction

Biopreservation represents a mild non-thermal food processing method that uses microorganisms and/or their metabolites to inhibit unwanted microorganisms without reducing nutritional or sensory properties in the food product [1]. Lactic acid bacteria (LAB) are promising candidates for seafood biopreservation due to their natural presence in fresh and minimally preserved seafood, including RTE meals like sushi and fish loins packed in vacuum and modified atmosphere [[2], [3], [4], [5], [6]]. Moreover, some preservation methods applied for mildly preserved seafood, such as salting, drying, smoking and vacuum packaging, can promote and select for LAB growth [7]. Thus, biopreservation can be combined with traditional (e.g. mild heat-treated, salting, smoking, chilling, modified atmosphere packaging) preservation methods in a hurdle technology approach [8] to produce minimally processed seafood to ensure microbiologically safe and stable products with high sensory and nutritional quality.

LAB are widely used in producing various fermented food products and have the status ‘generally recognized as safe’ (GRAS) in US [9], Qualified Presumption of Safety (QPS) in EU [10], and the government of Canada listed *Carnobacterium divergens* M35 as a permitted bioprotective culture in cold-smoked salmon (CSS) and trout (item No. C.1A) [11].

The LAB genera *Carnobacterium* and *Leuconostoc* are highly abundant in fish microbiota [2,[12], [13], [14]], are psychrotrophic and have shown promising results for biopreservation of seafood [[15], [16], [17]]. Strains representing both genera have demonstrated inhibitory effects against common spoilage bacteria as well as pathogens, including *Listeria monocytogenes* [5,[18], [19], [20], [21], [22]]. Major antimicrobial compounds produced by LAB include organic acids, ethyl alcohol, diacetyl, hydrogen peroxide, fatty acids, acetaldehyde, D-isomers of amino acids, CO_2_, as well as a variety of bacteriocins [23,24]. Bacteriocins are ribosomally synthesised peptides or proteins antagonistic to a narrow to broad spectrum of Gram-positive bacteria representing both spoilage bacteria and pathogens, such as *L. monocytogenes* [25]. Bacteriocins produced by LAB are divided into two main groups: class I-post-translationally modified bacteriocins, mainly lantibiotics - which contain lanthionine and class II-nonmodified heat-stable bacteriocins with four subclasses (IIa pediocin-like, IIb two-peptide, IIc cyclic bacteriocins and IId single linear peptides) [23]. Bacteriocins produced by *Leuconostoc* and *Carnobacterium,* belong to class II, active against *Listeria* [[26], [27], [28]]. Moreover, according to Ennahar Saïd et al. [26], class IIa bacteriocin-producing LAB are typically isolated from food sources such as fish, meat, and vegetables.

The differences between strains within the two genera are determined by diverse metabolic pathways depending on product type and storage conditions, although a limited effect on the sensory properties of food could be concluded [29]. Species in the genus *Leuconostoc* are facultatively anaerobic. A former growth kinetic experiment demonstrated a better growth adaptation in aerobic than anaerobic conditions; however, other process factors such as temperature, salt, and pH had a more decisive influence on the growth kinetic parameters of *Leuconostoc* [30]. In general, *Leuconostoc* metabolises citrate, d-lactate from glucose, produces CO_2_ from various carbon sources, and can display high tolerance to NaCl [[31], [32], [33]]. They are well known as contributors to the texture and taste of fermented food [34,35], as well as producers of biogenic amines (such as tyramine), dextran, and products related to buttery off-odours, such as diacetyl and acetoin [36,37].

Species from the *Carobacterium* genus are also frequently detected in raw seafood [38]. They can be described as ‘‘stress resilient’’ as they are fast growers, tolerant to high salt concentrations, oxygen, freezing, thawing, and high pressure [39,40], usually dominating in modified-atmosphere- [12,41,42], and vacuum packed products [43]. These characteristics have made *Carnobacterium* subject to extensive research on their application in different food products. Although reaching high concentrations, *Carnobacteria* will not necessarily affect the sensory properties of food negatively [44]. Moreover, food spoilage is highly dependent on bacterial interactions as well as interactions between bacteria and the food matrix [43].

Despite extensive research, the industrial application of LAB for biopreservation of seafood is minimal. Studies have focused on antimicrobial effects against specific target microorganisms such as *Listeria* spp. [6,[45], [46], [47], [48]], effects on growth in specific products such as CSS and shrimp [[49], [50], [51]], and effects on sensory properties [44,46,50,52]. In addition, different types of antimicrobial compounds were identified by applying the cell-free supernatants (CFS) of LAB against pathogenic and spoilage bacteria [53,54]. Without the growth of artificially added LAB strains, CFSs control microflora without affecting the sensorial and physicochemical properties of fresh fish which otherwise could be disturbed by high microbial growth during the storage period [55].

Few studies have explored LAB growth kinetic parameters under various processing scenarios, which should be investigated to apply biopreservation as a part of a hurdle technology approach for mildly processed seafood. The LAB strain's ability to grow and dominate the products in refrigerated conditions is critically important.

In a previous work, ten LAB strains (five *Carnobacterium* and five *Leuconostoc*) were isolated from RTE seafood products and selected as promising candidates for seafood biopreservation based on their high inhibitory effect against *Listeria* spp. and *Escherichia coli* [6]*. The present study aimed to i) investigate the antimicrobial mechanisms against selected pathogens, ii) investigate the strains' temperature-dependent growth kinetic parameters, iii) explore the process-dependent growth kinetic parameters relevant to the secondary prossessing of salmon, and iv) validate growth properties of selected strains in vacuum-packed fresh salmon at 4°C. To our knowledge, this is the first study to explore the potential of LAB for biopreservation of fresh pre-rigor filleted vacuum-packed salmon loins.*

## 2 Materials and methods

### 2.1 Lactic acid bacteria strains and experimental design

Ten LAB strains (*Carnobacterium maltaromaticum* 35 (C m.35), *C. maltaromaticum* 55 (C m.55), *C. maltaromaticum* 316 (C m.316), *C. maltaromaticum* 461 (C m.461), *C. divergens* 468 (C d.468), *Leuconostoc mesenteroides* 68 (Le.m.68), *L. citreum* (Le.c.105), *L. mesenteroides* 299 (Le.m.299), *L. lactis* 358 (Le.l.358), and *L. gelidum* 406 (Le.g.406)), previously isolated from sushi, cold-smoked salmon, and gravlax were included in the study based on their antimicrobial activity against pathogenic microorganisms commonly found in seafood products [6].

**Fig. 1:**
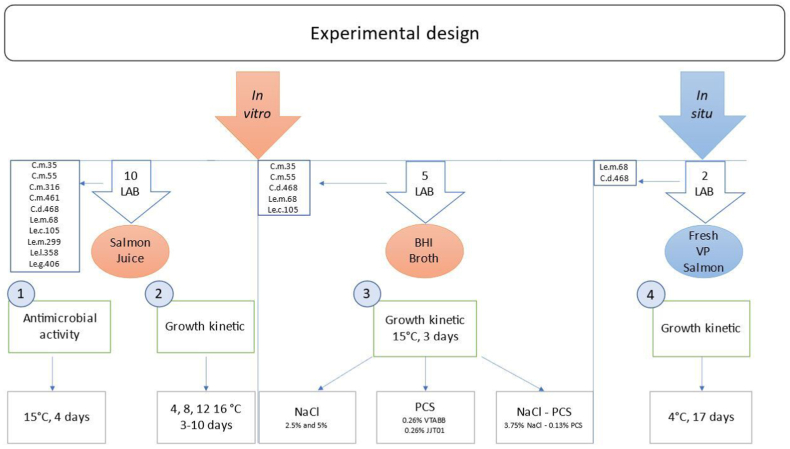
Experimental setup for [1] antimicrobial activity [2], temperature-dependent growth kinetics [3], process-dependent growth kinetics relevant to secondary processing of salmon, and [4] growth kinetics of selected strains in fresh vacuum packed (VP) salmon. Experiments 1, 2, and 3 are performed *in vitro* at 15 °C (except experiment 2- where the effect of different temperatures was tested). Abbreviations: C.m., *C.* maltaromaticum; C.d., *C. divergens;* L.m., *L. mesenteroides*; Le. c., *L. citreum*; Le. l., *L. lactis*; Le.g., *L. gelidum;* PCS, purified condensate smoke; VP, vacuum packed.

The experimental design was divided into four experiments (Fig. 1), of which three were performed *in vitro* (salmon juice or BHI), and the last was performed *in situ* (fresh salmon). The experiments were designed to study the strains’ 1) antimicrobial compounds, 2) temperature-dependent growth kinetics, 3) process-dependent growth kinetics relevant to the secondary processing of salmon, and 4) growth kinetic of two strains in vacuum-packed (VP) salmon stored at 4 °C. *In vitro* studies were performed at 15 °C, except for the temperature-dependent growth kinetics [2], where a range of different temperatures (4, 8, 12, 16 °C) was selected.

For *in vitro* experiments, the LAB strains were cultured on De Man, Rogosa and Sharpe (MRS) (Oxoid, Norway) or M17 medium (Oxoid, Norway) for 2–5 d, anaerobically (in anaerobic containers with GasPak EZ Anaerobe container system sachets w/indicator (BD, Norway) at 25 °C and precultured in MRS/M17 broth with 10% of lactose (Oxoid) (experiment 1 and 2) or in BHI broth (Oxoid, Norway) (experiment 3) for 24h at 15 °C. For *in situ* experiment, the LAB was cultured on MRS/M17 medium, with the same conditions as *in vitro*, and then one colony of each LAB was transferred to MRS/M17 broth for 2.5 d at 8 °C to adapt to cold storage. Prepared cultures were diluted to an optical density at 660 nm (OD660) of 0.22 (∼10^8^ CFU/ml).

### 2.2 Experiment 1: antimicrobial compounds of cell-free supernatant from LAB

#### 2.2.1 Salmon juice preparation

Sterile, supplemented (10 g/l of d-glucose, 100 ml/l of 1 M K_2_HPO4/KH_2_PO_4_ buffer solution, pH 6.7) salmon juice was prepared and used as a growth medium to mimic salmon's nutrient composition, as described in Stupar et al. (2021).

#### 2.2.2 Preparation of cell-free supernatants (CFS)

The LAB strains were prepared as described in 2.1. A volume of 300 μL of the diluted culture was then inoculated in 30 ml of either MRS or M17 broth and incubated at 15 °C for 96 h, for temperature adaption. Cell-free supernatants (CFS) were prepared as described by Yang et al. [56]. In brief, the cultures were centrifuged (4500×*g* for 10 min), and the supernatant was filtered through a 0.22 μm syringe filter (Chromatographic Specialties Inc, ON, Canada) (denoted untreated CFS). To obtain a neutralised supernatant (neutralised CFS), pH was adjusted to pH 6 with 1 M HCl/NaOH to remove the inhibitory effect of organic acids. Further, the supernatant was neutralised and treated with 1 mg/ml catalase (Sigma-Aldrich Corporation, USA) for 30 min at 25 °C to eliminate the possible inhibitory action of hydrogen peroxide (H_2_O_2_) (denoted as bacteriocin/bacteriocin-like substances (BLS)). All three supernatants (untereated CFS, neutralised CFS, and bacteriocin/BLS) were filtered again through the 0.22 μm syringe filter and frozen at −80 °C until use.

#### 2.2.3 Preparation of target strains

*L. innocua* (CCUG 15531) and *E. coli* (CCUG 38079), were grown on Brain Heart Infusion (BHI) agar (Oxoid) at 37 °C for 24 h, followed by the transfer of one colony to BHI broth (Oxoid) and incubation at 15 °C for 24 h.

#### 2.2.4 Inhibitory assay

The inhibitory assay was performed in a 96-well microplate setup at 15 °C for 96 h. Each well contained 196 μl of supplemented salmon juice and 2 μl of standardised LAB inoculums or CFS (untreated, neutralised and bacteriocin/BLS). The initial concentrations of bacteria in the assay were then 10^6^ CFU/ml for LAB and 10^2^ CFU/ml for the target organisms. Four parallel wells were set up for LAB monocultures, the co-cultures and cultures added cell-free supernatants, while eight parallels were used for target monocultures. Uninoculated salmon juice was applied as a negative control, while LAB monoculture and target monoculture were used as growth controls. For bacterial quantification, a serial dilution was performed followed by spotting of 5 μL of each dilution on target-selective agars: Violet Red Bile Agar (VRBA) (Oxoid, Norway), incubated at 37 °C for 24 h for *E. coli* and Brilliance *Listeria* Agar (BLA) (Oxoid, Norway) with Brilliance *Listeria* Selective Supplement (Oxoid, Norway), incubated at 37 °C for 24 h for *Listeria innocua*. LAB strains were quantified on MRS agar, anaerobically, in anaerobic containers with GasPak EZ Anaerobe container system sachets w/indicator (BD) at 25 °C for 2–5 d. The target strains were also quantified on BHI agar as a control at 37 °C for 24 h. Inhibition level was classified into five categories [6]: total inhibition (no growth of target strain), high inhibition (>6 logCFU/ml reduction of target strain), medium inhibition (3–6 logCFU/ml), low inhibition (<3 logCFU/ml) and no inhibition (no significant reduction of target strain).

### 2.3 Experiments 2 and 3: In vitro growth kinetic experiments

The LAB strains were prepared as described in 2.1. For each *in vitro* experiment, 300 μl of preculture was inoculated into 30 ml of salmon juice or BHI broth, to obtain a concentration of 10^6^ CFU/ml. Three independent parallels were made per strain (n = 3), and non-inoculated fish juice or BHI broth was used as a media control. A temperature-dependent growth experiment was performed for the ten strains at 4, 8, 12 and 16 °C in supplemented salmon juice. Furthermore, five strains (C.m.35, C.m.55, C.d.468, Le. m.68, and Le. c.105) were tested for their ability to grow at 15 °C at different NaCl concentrations (0.5, 2.5% and 5%), and in BHI containing PCS (SmokEz VTABB RA12012 (VTABB) or JJT01 30764575 (JJT01), purchased from Red Arrow™ (Manitowoc, WI, USA). The final concentration of 0.26% PCS represents the recommended maximum level (2.6 g/1 kg processed fish product) according to the manufacturer.

Furthermore, the same five strains were tested in a combination of NaCl (3.75%) and PCS (0.13% VTABB/JJT01). The pH was measured once a day (12 and 16 °C) or every second day (4 °C and 8 °C) in the salmon juice and on the first and the last day in BHI broth under different process parameters with a Testo 206 portable pH2 electrode (Testo, Germany). OD_600_ was measured regularly (one to seven times per day) with a UV spectrophotometer (Shimadzu UV 1800, Germany) until stationary phase was reached to estimate growth kinetic parameters. Non-inoculated fish juice or BHI broth was used as a media control.

### 2.4 Experiment 4: In situ growth kinetic experiment

#### 2.4.1 Sample preparation and packaging

Fresh *pre-rigor* filleted farmed Atlantic salmon loins (*Salmo salar*, Salma, Norway) were purchased from a local retailer. The salmon loins were cut into 30 ± 3 g pieces five days post-harvest, and the randomised pieces were placed on an absorbent pad (25 ml water capacity, Tommen Gram). Each piece was inoculated with 1% (v/w) LAB using a pipette and a sterile spreader to distribute the liquid on the surface. Both inoculated and uninoculated salmon pieces were air dried for 15–20 min before vacuum packaging (20 μm polyamide (PA)/70-μm polyethylene (PE) bag (120 9 80 mm, Star-Pack Productive, Boissy-l’Aillerie, France) with a Webomatic Supermax-C vacuum machine (Webomatic, Bochum, Germany). Air was evacuated to an end pressure of 10 mbar before sealing. All samples were stored at 4 ± 1 °C for 17 d. Sampling was done every 2–3 d (n = 3) during the storage period.

#### 2.4.2 Microbiological analysis

Ten grams of each salmon piece was mixed with 90 g of peptone water (1 g/l of peptone (Oxoid) and 8.5 g/l of NaCl (VWR, Belgium), and homogenised in a stomacher (IUL Masticator, Spain), and serially diluted (10-fold) before plating. Lyngby's Iron agar (Oxoid, Norway) supplemented with 0,04% l-cysteine (Sigma-Aldrich) was used for quantification of total aerobic counts and H_2_S -producing bacteria, and incubated at 22 °C for 72 ± 6 h [57], while MRS and M17 agar were used for quantification of LAB using anaerobic incubation at 25 °C for 2–5 d.

### 2.5 Calculations and statistical analysis

Log transformed bacterial concentrations were fitted to the primary model of Baranyi and Roberts [58], available in DMFit in ComBase (www.combase.cc) for estimation of the temperature dependent growth kinetic parameters.

Maximum growth rates obtained from the primary model were further modelled as a function of temperature using a secondary square-root model [59] (Equation (1)).

(1) $$\mu ={\left(b\;\left(T-\text{Tmin}\right)\right)}^{2}$$

where *b* is the slope of the regression line, *T* is the experimental temperature, and T*min* is the theoretical minimum temperature for growth.

The Statistical Package for the Social Science (IBM SPSS, version 28, Armonk, NY, USA) was used for all statistical analysis (including a one-way ANOVA, a two-way ANOVA, Tukey post hoc, and Idenpendent Samples T Test, (p = 0.05)). Pearson's correlation was calculated in the Experiment 4 to compare bacterial growth on Iron agar and the selective medium for LAB.

The pH reduction in the growth kinetic experiments (Experiment 2 and 3) was calculated by Equation (2):

(2) $$\Delta \text{pH}=pH_{i}-\text{pH}$$

where *ΔpH* represents the reduction of pH, i.e., difference between the average pH value of each LAB (n = 3) at the beginning of the experiment (*pH*_*i*_) and the average pH of LAB (n = 3) at the end of the experiment.

The level of growth inhibition was calculated by Equation (3):

(3) ΔlogCFU/mL=ẋlogCFU/mLtargetmonoculture-xlogCFU/mLtargetinco-culturewithLAB

where *Δlog CFU/mL* represents the inhibition level, i.e., difference between average concentration (log CFU/mL) of target cells grown in monoculture (n = 8) and concentration (log CFU/mL) of target obtained in co-culture with LAB or added supernatants i-iii (n = 4). Results are given in log CFU/mL with standard deviation.

## 3 Results and discussion

### 3.1 Experiment 1: The antimicrobial compounds of cell-free supernatant of LAB

The antimicrobial activity of ten LAB strains (five *Carnobacterium* spp. and five *Leuconostoc* spp.) and their cell-free supernatants (CFS) were tested against the target strains *L. innocua* and *E. coli* in a salmon juice model system to mimic the nutritional composition of salmon.

Quantification of target monocultures was performed in parallel on selective and general growth media to confirm the ability of the selective media to support the growth of target strains in potentially suboptimal conditions. For both target strains, there was no significant difference in the bacterial counts on selective and non-selective medium (unpaired *t*-test, p = 0.85 for *E. coli* and p = 0.36 for *L. innocua*), indicating no significant growth inhibition on selective media. The final cell concentrations of the LAB monocultures ranged from 8 to 10 log CFU/ml, in accordance with Stupar et al. (2021).

In co-culture with *L. innocua*, all LAB strains showed inhibitory activity (Table 1). Four *Carnobacterium* strains (C.m.35, C.m.316, C.m.461, and C.d.468) demonstrated total inhibition of *L. innocua,* and one strain (C.m. 55) showed high inhibition. All *Leuconostoc* strains inhibited the growth of *L. innocua*, ranging from medium to high inhibition, and total inhibition was observed for *Leuconostoc* strain Le.g.406. In most cases, a considerably lower inhibitory effect against *L. innocua* was achieved for CFSs (untreated, neutralised and bacteriocin or bacteriocin-like substances (BLS)) compared to the LAB-target strain co-cultures. The only exception was the untreated and neutralised CFS of two *Carnobacterium* strains (C.m.35 and C.d.468) with high inhibition. Moreover, the H_2_O_2_-free supernatant of these *Carnobacterium* strains showed medium inhibition of *L. inoccua* (4.8–5.8 log CFU/ml reduction), suggesting that the strains produce bacteriocins or BLS. No or low inhibition of *L. innocua* was observed for the CFS of the other LABs (no significant difference between treated and untreated target strain, one-way ANOVA, p < 0.001) (Table 1).

**Table 1:** Antimicrobial activity of LAB against *L. innocua* and *E. coli*. The results are described as Δlog CFU/mL±SD, i.e., the difference between the average concentration (log CFU/mL) of the target monoculture (n = 8) and the concentration (log CFU/mL) of the target obtained in co-culture with LAB (n = 4). Significant differences (calculated by one-way ANOVA, Tukey HSD, p < 0.05) between four groups (cell co-culture, untreated CFS, neutralised CFS, and H_2_O_2_-free CFS) of each strain are indicated by letters (^abc^) with corresponding p-value in the same row.

LAB Strain ID	LAB Genus	Target Strain	Cell co-culture	Cell free supernatant	Neutralised cell free supernatant (-acid)	H_2_O_2_-free supernatant (BLS)	p-value
C.m.35	Carnobacterium	*L. innocua*	9.7 ± 0.0^a^	7.1 ± 1.7^ab^	6.8 ± 1.9^b^	5.8 ± 0.4^b^	p = 0.008
		*E. coli*	3.5 ± 0.2^a^	−0.1 ± 0.2^b^	0.1 ± 0.1^b^	0.1 ± 0.1^b^	p < 0.001
C.m.55	*Carnobacterium*	*L. innocua*	6.8 ± 1.8^a^	1.2 ± 2.2^b^	0.1 ± 0.3^b^	0.4 ± 0.3^b^	p < 0.001
		*E. coli*	1.7 ± 0.1^a^	0.1 ± 0.1^bc^	−0.2 ± 0.1^c^	0.2 ± 0.2^b^	p < 0.001
C.m.316	*Carnobacterium*	*L. innocua*	9.5 ± 0.0^a^	0.3 ± 0.2^b^	0.4 ± 0.3^b^	0.2 ± 0.1^b^	p < 0.001
		*E. coli*	1.8 ± 0.1^a^	−0.0 ± 0.2^b^	0.1 ± 0.1^b^	0.1 ± 0.1^b^	p < 0.001
C.m.461	*Carnobacterium*	*L. innocua*	9.4 ± 0.0^a^	1.9 ± 0.3^b^	0.6 ± 0.3^c^	0.6 ± 0.1^c^	p < 0.001
		*E. coli*	3.1 ± 0.5^a^	−0.4 ± 0.2^b^	−0.3 ± 0.2^b^	−0.5 ± 0.2^b^	p < 0.001
C.d.468	*Carnobacterium*	*L. innocua*	9.1 ± 0.0^a^	6.4 ± 1.8^ab^	7.2 ± 2.1^ab^	4.8 ± 2.8^b^	p = 0.065
		*E. coli*	2.3 ± 0.2^a^	0.3 ± 0.2^b^	−0.1 ± 0.1^b^	0.2 ± 0.2^b^	p < 0.001
Le.m.68	*Leuconostoc*	*L. innocua*	3.9 ± 0.2^a^	0.8 ± 0.6^b^	0.5 ± 0.1^b^	0.7 ± 0.1^b^	p < 0.001
		*E. coli*	3.3 ± 0.3^a^	−0.0 ± 0.1^b^	−0.1 ± 0.1^b^	0.1 ± 0.1^b^	p < 0.001
Le.c.105	*Leuconostoc*	*L. innocua*	7.9 ± 1.9^a^	−0.5 ± 0.0^b^	1.6 ± 4.8^b^	0.1 ± 0.3^b^	p = 0.002
		*E. coli*	2.5 ± 0.7^a^	0.3 ± 0.1^b^	0.0 ± 0.1^b^	−0.2 ± 0.1^b^	p < 0.001
Le.m.299	*Leuconostoc*	*L. innocua*	4.8 ± 0.2^a^	0.2 ± 0.1^b^	0.1 ± 0.2^b^	0.1 ± 0.1^b^	p < 0.001
		*E. coli*	4.9 ± 0.2^a^	0.6 ± 0.4^b^	0.0 ± 0.4^b^	0.0 ± 0.2^b^	p < 0.001
Le.l.358	*Leuconostoc*	*L. innocua*	3.4 ± 0.1^a^	−0.4 ± 0.2^b^	−0.1 ± 0.3^b^	−0.4 ± 0.1^b^	p < 0,001
		*E. coli*	4.2 ± 0.7^a^	0.5 ± 0.1^b^	−0.3 ± 0.1^b^	−0.2 ± 0.2^b^	p < 0.001
Le.g.406	*Leuconostoc*	*L. innocua*	9.7 ± 0.0^a^	−0.1 ± 0.1^b^	−0.0 ± 0.1^b^	0.0 ± 0.1^b^	p < 0.001
		*E. coli*	1.5 ± 0.1^a^	0.2 ± 0.0^b^	0.2 ± 0.0^b^	−0.1 ± 0.1^c^	p < 0.001

The higher inhibition level observed for the two *Carnobacterium* strains (C.m.35 and C.d.468) in co-culture and for untreated- and neutralised-CFS compared to the BLS-CFS, indicate more than one antimicrobial mechanism of the strains, or relatively low concentrations of bacteriocin-like substances in the BLS-CFS, as proposed by Yang et al. [56].

For *E. coli*, a growth reduction ranging from low to medium inhibition (1.5–4.9 log CFU/ml) was observed following co-culture with the LAB strains. Moreover, the CFSs (all treatments) from the ten LAB strains had no or low inhibitory effect against *E. coli* (Table 1). A similar result was observed by Jonkuvienė et al. [60], where *E. coli* was more resistant to the antimicrobial activity of LAB than the Gram-positive target bacteria tested (*Bacillus subtilis*, *Bacillus cereus, L. monocytogenes*, and *Staphylococcus aureus)*. In the same study, CFS with native pH expressed antimicrobial activity against all target bacteria but after neutralization and addition of catalase, CFS maintained its activity only against *L. monocytogenes* [60]. In general, Gram-negative bacteria are declared as being more resistant to antimicrobial compounds due to their cell wall structure [61]. The inhibitory effect of LAB against *E. coli* observed in co-culture in the present study is likely due to nutrient competition between the strains, as LAB are generally recognized as strong competitors in heterogeneous food [62].

In total, two out of ten selected LAB strains possessed BLS producing properties. Both strains represent genus *Carnobacterium* but are isolated from different food sources (C.m.35 from gravlax, and C.d.468 from sushi) [6]. Thus, the prevalence of BLS activity was higher than in similar studies from different food sources [63,64], with a 0.3–14% prevalence. However, the strains in the present study were based on a pre-selection [6] that most likely affects the prevalence. Class II bacteriocin-producing LAB have previously been isolated from various food sources such as fish, meat, and vegetables [26]. Moreover, factors related to LAB strains (strain origin, growth rates, inoculum levels), media (pH, nutrient composition, NaCl, temperature), or the physiological state of target strains have been reported as decisive for bacteriocin activity and production [[65], [66], [67]]. Identifying antimicrobial compounds and the mode of action (competition/antimicrobial products) is crucial for strain for application in biopreservation of salmon products since different properties of the salmon products (e.g.type of packaging and competitive microbiota) can affect the production of antimicrobial compounds.

### 3.2 Experiment 2: Temperature-dependent growth kinetics of LAB in a salmon juice model system

Growth experiments with five *Leuconostoc* and five *Carnobacterium* strains were performed at four different temperatures (4, 8, 12 and 16 °C) to define the temperature-dependent growth kinetic parameters in a salmon juice model system.

**Fig. 2:**
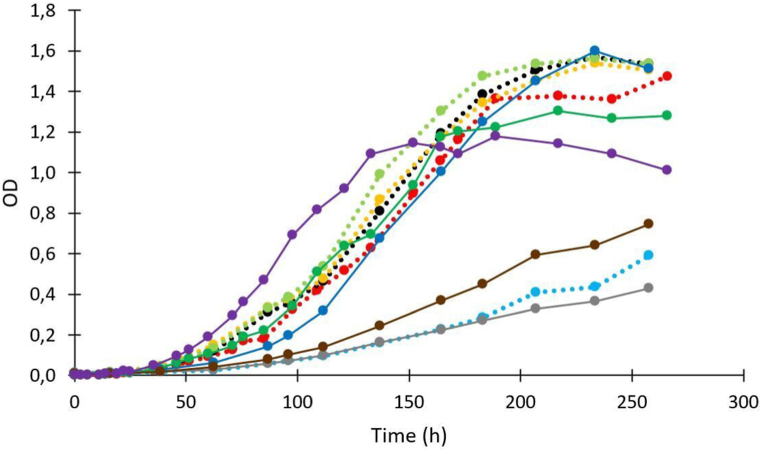
Growth of five *Leuconostoc* (solid lines: Le. m.68 , Le. c.105 , Le. m.299 , Le. l.358 , Le.g.406 ) and five *Carnobacterium* strains (dashed lines: C.m.35, C.m.55, C.m.316, C.m.461, C.d.468) at 4 °C as a function of time (hours).

All strains were able to grow at all temperatures with variations in growth rates, final concentrations, and lag phase (Table 2). However, a subset of three strains (Le.I.358, Le. c.105, and C.d.468) showed reduced growth rates at 4 °C (Table 2, Fig. 2). Also, three other strains (C.m.35, Le. m.299 and Le.g.406) grew at a significantly higher rate compared to the other strains at all temperatures (two-way ANOVA,p < 0.001) (Table 2).

**Table 2:** Growth kinetic parameters (lag phase (h), maximum growth rate *μ*_*max*_ (1/h), final concentration Ymax (log CFU/ml) for the selected LAB in fish juice (n = 3 ± SD) at different temperatures (4 °C, 8 °C, 12 °C and 16 °C). The parameters are estimated by primary model of Baranyi and Roberts (1994). R^2^, coefficient of determination; SE (fit), standard error of fit; NL, no lag phase. Significant differences for each temperature are indicated by letters (^abc^). The p-values for each temperature are shown at the bottom of the table. Roman numbers (^I/II/III^) indicate significant differences in species level with the corresponding p-value just below in the same box. Significant differences were calculated by two-way ANOVA (Tukey HSD).

Strain	T	Initial value	μ_max_	Y_max_	R^2^	SE	Lag phase
	*°C*	logCFU/ml	1/h	logCFU/ml			h
C.m.35	4	6.1 ± 0.08	0.03 ± 0.00^IIIb^	8.8 ± 0.06^IIb^	0.97	0.19	NL^Ia^
	8	6.3 ± 0.1	0.05 ± 0.00^IIa^	8.9 ± 0.07^IIef^	0.96	0.19	NL^Ia^
	12	6.3 ± 0.07	0.08 ± 0.00^Ia^	9.0 ± 0.04^Iab^	0.98	0.12	NL^Ia^
	16	6.7 ± 0.04	0.09 ± 0.00^Ia^	9.1 ± 0.02^Iab^	0.99	0.06	NL^Ia^
			p < 0.001	p < 0.001			
C.m.55	4	6.8 ± 0.06	0.02 ± 0.00^IVc^	9.0 ± 0.04^Ia^	0.99	0.08	NL^Ia^
	8	6.9 ± 0.17	0.03 ± 0.00^IIIb^	8.8 ± 0.09^I,II bc^	0.91	0.22	NL^Ia^
	12	6.7 ± 0.10	0.04 ± 0.00^IIb^	8.9 ± 0.06^IIbc^	0.98	0.10	2.3 ± 4.14^IIab^
	16	7.1 ± 0.09	0.05 ± 0.00^Ic^	8.9 ± 0.05^IIcd^	0.99	0.09	2.2 ± 2.85^IIbc^
			p < 0.001	p = 0.010			p = 0.001
C.m.316	4	6.9 ± 0.05	0.01 ± 0.00^IIIc^	9.0 ± 0.03^Ia^	0.99	0.08	NL^Ia^
	8	7.2 ± 0.10	0.02 ± 0.00^IIb^	9.0 ± 0.07^Iab^	0.96	0.14	NL^Ia^
	12	6.3 ± 0.13	0.05 ± 0.00^Ib^	8.8 ± 0.08^IIIe^	0.97	0.16	NL^Ia^
	16	7.0 ± 0.08	0.05 ± 0.00^Ic^	8.9 ± 0.06^IIe^	0.98	0.11	NL^Ia^
			p < 0.001	p < 0.001			
C.m.461	4	6.8 ± 0.04	0.02 ± 0.00^IVc^	9.0 ± 0.02^Ia^	0.99	0.06	NL^Ia^
	8	6.9 ± 0.13	0.02 ± 0.00^IIIb^	9.0 ± 0.08^Iab^	0.94	0.18	NL^Ia^
	12	6.3 ± 0.11	0.05 ± 0.00^IIb^	8.8 ± 0.08^IIId^	0.98	0.14	NL^Ia^
	16	6.9 ± 0.06	0.06 ± 0.00^Ic^	8.9 ± 0.05^IId^	0.99	0.08	NL^Ia^
			p < 0.001	p < 0.001			
C.d.468	4	6.8 ± 0.07	0.01 ± 0.00^IIe^	8.6 ± 0.06^IId^	0.98	0.08	12 ± 13.5^IIa^
	8	6.9 ± 0.05	0.02 ± 0.00^IIb^	9.0 ± 0.04^Icd^	0.99	0.07	NL^Ia^
	12	6.9 ± 0.1	0.03 ± 0.00^Ib^	9.0 ± 0.07^Ibc^	0.98	0.1	2.2 ± 4.67^Iab^
	16	6.9 ± 0.08	0.05 ± 0.00^Ic^	8.9 ± 0.07^Ie^	0.98	0.11	NL^Ia^
			p < 0.001	p < 0.001			p = 0.013
Le.m.68	4	6.9 ± 0.04	0.01 ± 0.00^IVcd^	9.0 ± 0.02^IIa^	0.99	0.05	10 ± 5.7^IIa^
	8	6.9 ± 0.04	0.02 ± 0.00^IIIb^	9.1 ± 0.03^Ia^	0.99	0.06	NL^Ia^
	12	6.8 ± 0.02	0.04 ± 0.00^IIb^	9.1 ± 0.0137^Ia^	0.99	0.02	8.8 ± 0.7^I,IIbc^
	16	6.9 ± 0.03	0.08 ± 0.00^Iab^	9.1 ± 0.01^Ia^	0.99	0.03	2.3 ± 0.6^Ibc^
			p < 0.001	p < 0.001			p = 0.012
Le.c.105	4	6.8 ± 0.06	0.01 ± 0.00^IVe^	8.4 ± 0.04^IVe^	0.98	0.07	9.3 ± 11.95^Ia^
	8	6.9 ± 0.05	0.02 ± 0.00^IIIb^	8.9 ± 0.05^IIde^	0.99	0.07	NL^Ia^
	12	6.6 ± 0.03	0.05 ± 0.00^IIb^	8.9 ± 0.0355^IIIcd^	0.99	0.05	27 ± 1.5^IId^
	16	7.1 ± 0.03	0.07 ± 0.00^Ibc^	9.0 ± 0.02^Ic^	0.99	0.03	2.8 ± 0.89^Ic^
			p < 0.001	p < 0.001			p = 0.004
Le.m.299	4	6.2 ± 0.07	0.03 ± 0.00^IIIb^	8.8 ± 0.06^IIb^	0.97	0.17	NL^Ia^
	8	6.2 ± 0.11	0.05 ± 0.00^IIa^	8.9 ± 0.08^IIefg^	0.96	0.22	NL^Ia^
	12	6.3 ± 0.07	0.08 ± 0.00^Ia^	9.0 ± 0.05^IIab^	0.98	0.12	NL^Ia^
	16	6.8 ± 0.03	0.09 ± 0.00^Ia^	9.0 ± 0.02^Ib^	0.99	0.05	NL^Ia^
			p < 0.001	p < 0.001			
Le.l.358	4	6.8 ± 0.04	0.01 ± 0.00^IIIde^	8.7 ± 0.04^IVc^	0.99	0.06	NL^Ia^
	8	6.9 ± 0.06	0.02 ± 0.00^IIIb^	8.8 ± 0.05^IIIg^	0.98	0.09	NL^Ia^
	12	6.9 ± 0.04	0.04 ± 0.00^IIb^	8.9 ± 0.03^IId^	0.99	0.04	11 ± 1.7^Ic^
	16	6.8 ± 0.07	0.07 ± 0.00^Ibc^	8.9 ± 0.04^Ie^	0.99	0.07	0.3 ± 1.7^Iab^
			p < 0.001	p < 0.001			p = 0.171
Le.g.406	4	6.1 ± 0.07	0.04 ± 0.00^IIIa^	8.8 ± 0.05^IIb^	0.98	0.16	NL^Ia^
	8	6.2 ± 0.08	0.05 ± 0.00^IIa^	8.8 ± 0.05^IIfg^	0.98	0.15	NL^Ia^
	12	6.3 ± 0.06	0.08 ± 0.00^Ia^	8.9 ± 0.04^Icd^	0.99	0.10	NL^Ia^
	16	6.7 ± 0.03	0.08 ± 0.00^Ia^	8.9 ± 0.02^Id^	0.99	0.05	NL^Ia^
			p < 0.001	p < 0.001			
			p < 0.001				
p-value	4			p < 0.001			p = 0.048
	8		p < 0.001	p < 0.001			p = 0.038
	12		p < 0.001	p < 0.001			p < 0.001
	16		p < 0.001	p < 0.001			p < 0.001

**Fig. 3:**
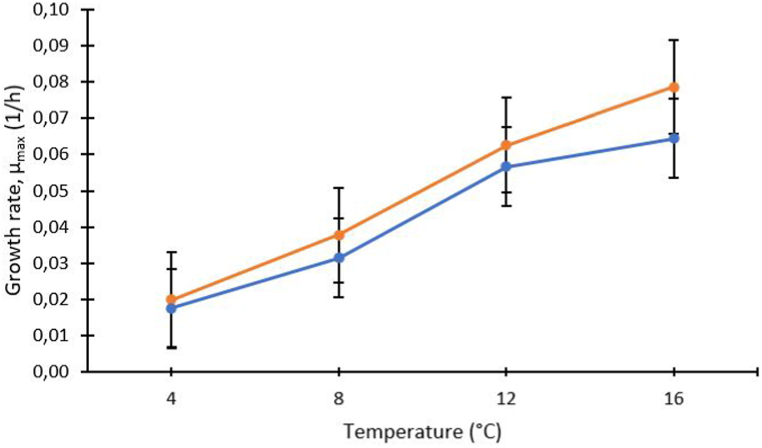
Estimated marginal means of maximum growth rate (*μmax*) of five *Carnobacterium* strains  and five *Leuconostoc* strains  at four temperatures (4, 8, 12 and 16 °C). Each point represents the mean (n = 15) of five strains (n = 3 for each strain), and vertical bars indicate ± SE, calculated by a two-way ANOVA.

The ability of LAB strains to grow at 4 °C (Fig. 2) without a significant lag phase (seven out of ten strains displayed no lag phase) is particularly interesting for application in the biopreservation of refrigerated fresh and processed salmon products. It must be emphasised that the LAB strains were precultured in BHI broth at 15 °C to prevent temperature stress due to chilling, as the lag time depends on actual as well as previous environmental conditions and the physiological status of the cell [68]. At the genus level, there was no significant difference between *Carnobacterium* and *Leuconostoc* strains with respect to μ_*max*_ (Fig. 3) and lag phase at 4 °C. However, a significant difference in the final cell concentration was observed at the same temperature (two-way ANOVA, p = 0.004) (data not shown).

Furthermore, tha fact that no lag phase was observed for any LAB strain grown at 8 °C must be emphasised in the case of temperature abuse and the ability of LAB to spoil food. The observed results (Table 2) present strain-to-strain variations in temperature response and demonstrate that temperature firmly controls the growth parameters [69,70].

A Ratkowsky square root model [59] was used to describe *μ*_*max*_ as a function of temperature. This model has been proven valid and better in the interpretation of data than the Arrhenius type model [70], and the study of Hoel et al. [71] confirmed that the model could describe the effect of temperature on the growth of LAB. The slope of the regression line (b) was numerically higher for Le. c.105 and Le. m.68, suggesting a stronger temperature response, however not significantly different from Le. l.358, C.m.35, C.d.468, and Le. m.299 (Table 3).

**Table 3:** Parameters of the secondary square-root type model for the effect of temperature on the growth rates of lactic acid bacteria (LAB), where *b* is the slope of the regression line, *Tmin* is the theoretical minimum temperature for growth and *R*^*2*^ represents the fit of the model. Significant differences between strains are calculated by one-way ANOVA (Tukey HSD) and indicated by letters (^abcd^).

Strain	b	T_min_ (°C)	R^2^
C.m.35	0.012 ± 0.001^abc^	−10.3 ± 1.7	0.93
C.m.55	0.009 ± 0.001^d^	−10 ± 1.1	0.99
C.m.316	0.010 ± 0.000^bcd^	−8.6 ± 0.2	0.90
C.m.461	0.009 ± 0.000^cd^	−9.9 ± 0.7	0.95
C.d.468	0.011 ± 0.001^abcd^	−5.1 ± 1.0	0.97
Le.m.68	0.013 ± 0.000^a^	−5.3 ± 0.1	0.99
Le.c.105	0.014 ± 0.001^a^	−3.2 ± 0.7	0.97
Le.m.299	0.011 ± 0.000^abcd^	−11.5 ± 1.1	0.88
Le.l.358	0.013 ± 0.000^ab^	−4.2 ± 0.5	0.97
Le.g.406	0.009 ± 0.001^d^	−17.9 ± 2.2	0.90

This model could serve as a starting point for the prediction of growth response following temperature abuse or for using LAB in some non-cold stored products, while obtained minimum temperatures can predict their use for application in combination with preservation methods such as superchilling. However, regarding a more complex system such as a real product, not all limiting factors could be included by predictive mathematical modelling, so it must be validated by challenge testing [72].

The pH measurements were done during the temperature-dependent growth experiment (Table 4). A strong negative correlation (r=(−0.91)-(-0.96), p < 0.001) was found between changes in pH (ΔpH) and growth (OD_600_) at all temperatures. A pH drop was observed at cell densities >7 log CFU/ml, which is in accordance with other studies [69,73,74]. Thus, the pH drop can be used as a parameter indicating the bacterial growth stage [69]. The highest decrease in pH was found at 4 °C, except for the three strains (C.m.468, Le. c.105, and Le. l.358), which did not reach the stationary phase at 4 °C.

**Table 4:** Reduction of pH at 4, 8, 12 and 16 °C in fish juice. The results are presented as Δ_pH_ values.

Strain	Growth temperature (°C)				
	4	8	12	16	
	ΔpH	ΔpH	ΔpH	ΔpH	p-value
C.m.35	1.4 ± 0.008^Ie^	1.3 ± 0.009^IIe^	1.2 ± 0.004^IIId^	1.2 ± 0.0^IIIbc^	p < 0.001
C.m.55	1.1 ± 0.07^Ic^	0.6 ± 0.03^IIa^	0.7 ± 0.05II^bc^	1 ± 0.01^Ia^	p < 0.001
C.m.316	1.1 ± 0.04^Ic^	0.76 ± 0.02^IIb^	0.6 ± 0.04^IIIab^	1 ± 0.01^Ia^	p < 0.001
C.m.461	1.2 ± 0.03^Id^	0.86 ± 0.02^IIc^	0.77 ± 0.01^IIIc^	1.2 ± 0.004I^bc^	p < 0.001
C.d.468	0.46 ± 0.04^IIIa^	1 ± 0.008^Id^	0.76 ± 0.008^IIc^	1 ± 0.0^Ia^	p < 0.001
Le.m.68	1.5 ± 0.02^If^	1.4 ± 0.01^IIf^	1.4 ± 0.05^IIe^	1.2 ± 0.01^IIcd^	p < 0.001
Le.c.105	0.4 ± 0.02^IVa^	0.8 ± 0.01^IIb^	0.5 ± 0.01^IIIa^	1.2 ± 0.02^Ib^	p < 0.001
Le.m.299	1.4 ± 0.02^Ie^	1.3 ± 0.004^IIe^	1.2 ± 0.0^IIIde^	1.2 ± 0.004^IIIbcd^	p < 0.001
Le.l.358	0.6 ± 0.02^II,IIIb^	0.6 ± 0.02^IIIa^	0.7 ± 0.03^IIc^	1.2 ± 0.01^Ibcd^	p < 0.001
Le.g.406	1.4 ± 0.01^Ie^	1.3 ± 0.01I^IIef^	1.3 ± 0.0^IIIde^	1.3 ± 0.0^IIId^	p < 0.001
	p < 0.001	p < 0.001	p < 0.001	p < 0.001	

The formula Δ_pH_ = pH_i_-pH was used to obtain Δ_pH_ values where Δ_pH_ represents the reduction of pH, i.e., the difference between the average pH value of each LAB (n = 3) at the beginning of the experiment (pH_i_) and the average pH of LAB (n = 3) at the end of the experiment. The letters ^abcd^ indicate significant differences for each temperature with corresponding p-value at the bottom of the table. Roman numbers (^I/II/III^) indicate significant differences in species level with the corresponding p-value just below in the same box. Significant differences were calculated by two-way ANOVA (Tukey HSD).

### 3.3 Experiment 3: Process-dependent growth kinetics of LAB in BHI broth

Based on high inhibitory activities towards selected pathogens [6] (Table 1) and/or their high growth rates at low temperatures (Table 2), five LABs (C.m.35, C.m.55, C.d.468, Le. m.68 and Le. c.105) were selected for testing of tolerance to relevant process parameters for secondary processing of salmon. A commercially important RTE salmon product is cold-smoked salmon (CSS). Production of CSS includes several processing steps including salting (<6.0% in the water phase), dehydration and wood smoking at 25–30 °C. Alternatively, artificial smoke flavouring can be used by applying atomised purified condensed smokes (PCS) [75]. As PCSs are considered healthier than traditional wooden smoke [76], we focused on NaCl and PCS as process parameters.

#### 3.3.1 Sodium chloride (NaCl)

The effect of NaCl (0.5–5%) was studied in BHI broth at 15 °C. All strains were able to grow at the NaCl concentrations tested, with variations in μ_max_, Y_max_, and lag phase among tested strains (Table 5). In general, growth rates and final cell concentrations were reduced with increasing salt concentrations, except for the two *Leuconostoc* strains, where the growth rates were not affected by 2.5% NaCl in the medium (Table 5). Thus, the strain's growth properties are compatible with application in CSS, in which 2.5–3.5% (w/w) salt content is normally used to inhibit undesirable microbiota [77]. Furthermore, no differences in the final pH were observed for strains grown in 2.5% NaCl compared to the unsupplemented control, except for the strains C.d.468 (ΔpH = 0.5 ± 0.005) and Le. m.68 (ΔpH = 0.44 ± 0.005), where pH was significantly reduced compared to the control (p < 0.001). At 5% NaCl, the final pH was significantly reduced in all supplemented media compared to the control, except for the strains C.m.35 and C.m.55. The strains C.d.468 (ΔpH = 0.6 ± 0.005) and Le. m.68 (ΔpH = 0.6 ± 0.0) performed the highest reductions.

**Table 5:** Growth kinetic parameters (lag phase (h), maximum growth rate μ_max_ (1/h), final concentration Ymax (log CFU/ml) for the selected LAB at 0.5% (control), 2.5% (n = 3), 5% (n = 3) in BHI broth at 15 °C. LAB without salt addition was used as control (n = 3). The parameters are estimated by primary model of Baranyi and Roberts (1994). R^2^-coefficient of determination; SE (fit), standard error of fit; NL, no lag phase; NG, negligible lag phase. Significant differences for each NaCl concentration are indicated by letters (^abc^). The p-values for each NaCl concentration are shown at the bottom of the table. Roman numbers (^I/II/III^) indicate significant differences in species level with the corresponding p-value just below in the same box. Significant differences were calculated by one-way ANOVA (Tukey HSD).

Strain	NaCl	Initial value	μ_max_	Y_max_	R^2^	SE	Lag phase
		logCFU/ml	1/h	logCFU/ml			h
C.m.35	0.5%	6.3 ± 0.06	0.12 ± 0.006^Icd^	9.2 ± 0.02^Iab^	0.99	0.06	1.7 ± 0.9^IIa^
	2.5%	6.3 ± 0.06	0.07 ± 0.003^IIc^	9.2 ± 0.03^IIa^	0.99	0.08	NL^Ia^
	5%	6.8 ± 0.05	0.04 ± 0.002^IIId^	8.9 ± 0.04^IIIb^	0.99	0.06	4.4 ± 2.1^IIIb^
			p < 0.001	p < 0.001			p < 0.001
C.m.55	0.5%	6.3 ± 0.07	0.11 ± 0.006^Id^	9.3 ± 0.0286^Ia^	0.99	0.07	1.4 ± 1^Ia^
	2.5%	6.3 ± 0.06	0.08 ± 0.003^IIc^	9.2 ± 0.03^IIa^	0.99	0.08	NG^Ia^
	5%	6.8 ± 0.03	0.04 ± 0.001^IIIc^	9.0 ± 0.02^IIIa^	0.99	0.04	6.3 ± 1.3^IIc^
			p < 0.001	p < 0.001			p < 0.001
Le.m.68	0.5%	6.3 ± 0.06	0.15 ± 0.007^Ia^	8.9 ± 0.03^Ic^	0.99	0.08	NL^Ia^
	2.5%	6.7 ± 0.05	0.15 ± 0.01^Ia^	8.8 ± 0.02^IIc^	0.99	0.06	4.8 ± 0.8^IId^
	5%	6.5 ± 0.06	0.08 ± 0.006^IIa^	8.6 ± 0.04^IIId^	0.99	0.08	5.1 ± 1.6^IIbc^
			p < 0.001	p < 0.001			p < 0.001
Le.c.105	0.5%	6.1 ± 0.04	0.14 ± 0.006^Ib^	8.8 ± 0.01^Ic^	0.99	0.05	NL^Ia^
	2.5%	6.3 ± 0.03	0.13 ± 0.00577^Ib^	8.8 ± 0.01^IIc^	0.99	0.03	1.9 ± 0.5^IIb^
	5%	6.8 ± 0.03	0.07 ± 0.003^IIb^	8.7 ± 0.01^IIIc^	0.99	0.04	4.2± 1^IIIb^
			p < 0.001	p < 0.001			p < 0.001
C.d.468	0.5%	6.2 ± 0.06	0.13 ± 0.006^Ibc^	9.2 ± 0.03^Ib^	0.99	0.07	2.1 ± 0.9^I,IIa^
	2.5%	6.6 ± 0.06	0.08 ± 0.004^IIc^	9.15 ± 0.03^IIb^	0.99	0.07	3.9 ± 1.4^IIc^
	5%	6.4 ± 0.01	0.04 ± 0.0006^IIIe^	9.01 ± 0.05^IIIab^	0.99	0.03	NG^Ia^
			p < 0.001	p < 0.001			p = 0.014
p-value	0.5%		p < 0.001	p < 0.001			p = 0.053
	2.5%		p < 0.001	p < 0.001			p < 0.001
	5%		p < 0.001	p < 0.001			p < 0.001

Generally, 1–2% NaCl is optimal for LAB growth, while reduction/inhibition of the growth of most LAB strains has been observed above 3% NaCl [[78], [79], [80]]. However, salt-tolerant species such as *Tetragenococcus halophilus* are important for high-salt products such as soy sauce [81]. In our study, all tested strains grew at 5% NaCl, with a two to three-times reduction of growth rates compared to controls (0.5% NaCl). It is previously shown that LAB strains isolated from CSS were able to grow at 5% w/v of NaCl and 5 °C [82], while Connil et al. [83] demonstrated that a *Carnobacterium* strain isolated from trout viscera could grow at even more strenuous conditions (6.5% NaCl and 3 °C) in a sterile CSS extract. Although the selected strains (with and without BLS activity) in the present study have potential for use in salted-type products, further studies are required to determine the *anti*-listerial activity/BLS production of the strains in high salt food products.

#### 3.3.2 Purified condensed smoke (PCS)

The effects of two PCSs (VTABB and JJT01) were tested at a concentration of 0.26% in BHI at 15 °C. This concentration represents the maximum level of smoke condensate that can be used for fish products, according to the manufacturer. The TPC of 0.26% VTABB and JJTO1 were 10.9 ± 0.003 mg and 4.7 ± 0.002 mg/100 ml, respectively [84].

All tested strains could grow in the presence of both types of PCSs, with observed differences among strains (Table 6), and without significant changes in pH, compared to the control (BHI, no added PCS). PCS significantly reduced the μ_max_ (one-way ANOVA, p < 0.001) of all strains compared to controls; however, different resistance of strains was observed for the two types of PCS. No differences (C.m.55 and C.d.468), or higher sensitivity to VTABB than JJT01 (except for Le. m.68) were observed (Table 6). In the study of Lee et al. [84], all *Aeromonas* strains were completely inhibited by VTABB and mostly inhibited by JJTO1 at the same PCS concentration, and differences can probably be attributed to the two times higher TPC of VTABB than JJT01 [84]. Variations in the antimicrobial activity of PCS due to component differences were also suggested by Takeda et al. [85]. In addition, the antimicrobial effect of phenolic compounds also depends on factors such as temperature, pH and a_w_.

**Table 6:** Growth kinetic parameters (lag phase (h), maximum growth rate, μ_max_ (1/h), final concentration Y_max_ (log CFU/ml) for the selected LAB at 0.26% VTABB (n = 3), and 0.26% JJTO1 (n = 3) in BHI broth at 15 °C. LAB without liquid smoke addition was used as control (n = 3). The parameters are estimated by primary model of Baranyi and Roberts (1994). R^2^-coefficient of determination; SE (fit), standard error of fit; NL, no lag phase. Significant differences for each PCS concentration and control are indicated by letters (^abc^). The p-values for each PCS concentration and control are shown at the bottom of the table. Roman numbers (^I/II/III^) indicate significant differences in species level with the corresponding p-value just below in the same box. Significant differences were calculated by one-way ANOVA (Tukey HSD).

Strain	PCS	Initial value	μ_max_	Y_max_	R^2^	SE	Lag phase
	0.26%	logCFU/ml	1/h	logCFU/ml			h
C.m.35	control	6.6 ± 0.09	0.1 ± 0.008^Ic^	9.2 ± 0.06^IIa^	0.98	0.14	NL^Ia^
	VTABB	6.2 ± 0.08	0.04 ± 0.002^IIId^	9.4 ± 0.54^Ia^	0.97	0.16	NL^Ia^
	JJTO1	6.1 ± 0.1	0.06 ± 0.005^IIc^	9.2 ± 0.08^IIa^	0.98	0.13	0.6 ± 3.5^Ia^
			p < 0.001	p < 0.001			p = 0.325
C.m.55	control	6.2 ± 0.02	0.12 ± 0.002^Ib^	9.2 ± 0.01^Ia^	0.99	0.04	NL^Ia^
	VTABB	6.0 ± 0.06	0.08 ± 0.003^IIb^	9.1 ± 0.05^IIb^	0.99	0.1	NL^Ia^
	JJTO1	7.1 ± 0.04	0.07 ± 0.004^IIb^	9.2 ± 0.02^IIa^	0.99	0.05	8.6 ± 1.2^IIb^
			p < 0.001	p < 0.001			p < 0.001
Le.m.68	control	6.3 ± 0.03	0.15 ± 0.004^Ia^	8.8 ± 0.01^IIb^	0.99	0.04	NL^Ia^
	VTABB	5.9 ± 0.04	0.13 ± 0.004^IIa^	8.8 ± 0.02^IIc^	0.99	0.06	NL^Ia^
	JJTO1	6.8 ± 0.04	0.09 ± 0.004^IIIa^	8.9 ± 0.02^Ic^	0.99	0.06	NL^Ia^
			p < 0.001	p = 0.002			p = 0.422
Le.c.105	control	6.8 ± 0.04	0.1 ± 0.006^Ic^	8.8 ± 0.01^Ib^	0.99	0.05	3.6 ± 0.8^IIb^
	VTABB	6.1 ± 0.17	0.06 ± 0.009^IIIc^	8.7 ± 0.14^IIc^	0.92	0.29	NL^Ia^
	JJTO1	6.2 ± 0.09	0.1 ± 0.007^IIa^	8.8 ± 0.06^Id^	0.97	0.15	NL^Ia^
			p < 0.001	p = 0.008			p < 0.001
C.d.468	control	6.4 ± 0.05	0.11 ± 0.004^Ic^	9.2 ± 0.02^Ia^	0.99	0.08	NL^Ia^
	VTABB	6.0 ± 0.05	0.07 ± 0.002^IIb^	9.1 ± 0.04^IIIb^	0.99	0.09	NL^Ia^
	JJTO1	7.0 ± 0.01	0.08 ± 0.001^IIb^	9.17 ± 0.008^IIb^	0.99	0.02	7.1 ± 0.4^IIb^
			p < 0.001	p < 0.001			p < 0.001
p-value	control		p < 0.001	p < 0.001			p = 0.010
	VTABB		p < 0.001	p < 0.001			
	JJTO1		p < 0.001	p < 0.001			p < 0.001

#### 3.3.3 Combined effects of NaCl and PCS

The strains' ability to grow when combining 3.75% NaCl and 0.13% PCS (VTABB or JJTO1) was assessed in BHI at 15 °C. The TPC of 0.13% VTABB and JJTO1 were 5.45 ± 0.003 mg/100 ml and 2.35 ± 0.002 mg/100 ml, respectively [84]. The BHI broth without supplemented NaCl and PCS was used as a control.

The growth rates of all strains (Table 7) were higher in broth combining NaCl and JJTO1 than NaCl and VTABB (one-way ANOVA, p < 0.001). Although PCSc and NaCl concentrations were lower than in the previous studies (3.3.1 and 3.3.2), growth rates were almost two to four times lower than control, except for *Leuconostoc* strains, where rates were numerically higher but significantly different from the control. No differences in final cell concentrations were observed when comparing the control and supplemented BHI for the strains C.m.35 and C.m.55.

**Table 7:** Growth kinetic parameters (lag phase (h), maximum growth rate, μ_max_ (1/h), final concentration Y_max_ (log CFU/ml) for the selected LAB at 0.13% VTABB and 3.75% NaCl (n = 3), 0.13% JJTO1 and 3.75% NaCl (n = 3) in BHI broth at 15 °C. BHI without PCS and salt addition were used as control (n = 3). The parameters are estimated by primary model of Baranyi and Roberts (1994). R^2^-coefficient of determination; SE (fit), standard error of fit; NL, no lag phase; NG, negligible lag phase. Significant differences for each PCS/salt concentration and control are indicated by letters (^abc^). The p-values for each PCS/salt concentration and control are shown at the bottom of the table. Roman numbers (^I/II/III^) indicate significant differences in species level with the corresponding p-value just below in the same box. Significant differences were calculated by one-way ANOVA (Tukey HSD).

Strain	PCS + Salt	Initial value	μ_max_	Y_max_	R2	SE	Lag phase
	0.13% + 3.75%	logCFU/ml	1/h	logCFU/ml			h
C.m.35	control	6.3 ± 0.04	0.12 ± 0.003^Ib^	9.3 ± 0.01^Ia^	0.99	0.04	1.3 ± 0.6^Ib^
	VTABB + NaCl	6.2 ± 0.05	0.03 ± 0.001^IIId^	9.2 ± 0.7^Ia^	0.99	0.05	1.2 ± 2.7^Ia^
	JJTO1+NaCl	6.4 ± 0.06	0.05 ± 0.003^IIcd^	9.1 ± 0.06^Ia^	0.99	0.07	1.8 ± 2.3^Ia^
			p < 0.001	p = 0.534			p = 0.658
C.m.55	control	6.3 ± 0.04	0.11 ± 0.004^Ibc^	9.3 ± 0.02^Ia^	0.99	0.04	1.3 ± 0.7^Ib^
	VTABB + NaCl	6.2 ± 0.06	0.03 ± 0.002^IIId^	9.08 ± 1.3^Ia^	0.99	0.07	5.7 ± 3.6^IIa^
	JJTO1+NaCl	6.3 ± 0.06	0.06 ± 0.003^IIc^	9.1 ± 0.05^Ia^	0.99	0.07	1.6 ± 2.2^Ia^
			p < 0.001	p = 0.376			p = 0.002
Le.m.68	control	6.3 ± 0.03	0.15 ± 0.004^Ia^	8.8 ± 0.01^Id^	0.99	0.0471	NG^Iab^
	VTABB + NaCl	6.6 ± 0.03	0.08 ± 0.002^IIIa^	8.7 ± 0.01^IIIa^	0.99	0.0403	3.9 ± 0.8^IIa^
	JJTO1+NaCl	6.4 ± 0.04	0.08 ± 0.003^IIb^	8.8 ± 0.02^IIc^	0.99	0.0625	NL^I,IIa^
			p < 0.001	p < 0.001			p = 0.021
Le.c.105	control	6.5 ± 0.02	0.12 ± 0.002^Ib^	8.9 ± 0.009^Ic^	0.99	0.02	NG^Iab^
	VTABB + NaCl	6.0 ± 0.06	0.05 ± 0.002^IIIb^	8.7 ± 0.05^IIIa^	0.99	0.08	NL^Ia^
	JJTO1+NaCl	6.4 ± 0.06	0.1 ± 0.006^IIa^	8.8 ± 0.03^IIc^	0.99	0.06	3 ± 1.2^IIa^
			p < 0.001	p < 0.001			p < 0.001
C.d.468	control	6.4 ± 0.05	0.11 ± 0.004^Ic^	9.2 ± 0.02^Ib^	0.99	0.08	NL^Ia^
	VTABB + NaCl	7.1 ± 0.1	0.04 ± 0.007^IIIc^	8.9 ± 0.1^IIIa^	0.93	0.19	18.6 ± 4.8^IIb^
	JJTO1+NaCl	6.6 ± 0.05	0.05 ± 0.002^IId^	8.9 ± 0.03^IIb^	0.99	0.06	4.7 ± 1.7^Ia^
			p < 0.001	p < 0.001			p = 0.004
p-value	control		p < 0.001	p < 0.001			p = 0.013
	VTABB + NaCl		p < 0.001	p = 0.047			p < 0.001
	JJTO1+NaCl		p < 0.001	p < 0.001			p = 0.082

The pH reduction following growth did not differ between the two PCS combinations for the strains Le. m.68, C.d.468 and Le. c.105, although it differed from the control (p < 0.001). The highest pH reduction was performed by the strains Le. m.68 (ΔpH = 0.6 ± 0.0) and C.d.468 (ΔpH = 0.5 ± 0.005), while the lowest was performed by the strains C.m.55 (ΔpH = 0.17 ± 0.00) and C.m.35 (ΔpH = 0.26 ± 0.005) in VTABB-NaCl tubes, where the reduction was even lower than in the control (p < 0.001).

The observed LAB growth kinetics at the different process parameters indicate that the PCS JJT01 is most suitable for application with LAB, alone and in combination with NaCl. This might be due to chemical differences in the two tested PCSs, resulting in different sensorial and antimicrobial effects on the food product [86,87]. This chemical composition is reflected in different proportions of organic acids, phenolic and carbonyl components, determined by the type, wood moisture content and temperature pyrolysis [88]. In general, resistance to PCS and NaCl could indicate the potential of applying these LAB strains for long-storage products, as highlighted by Aymerich et al. [65]. Strain to strain variations in tolerance to PCS and NaCl could be caused by different responses/damages to cell structures [85]. Regardless, LAB supplemented with VTABB did not express a lag phase, while a prolonged lag phase was found in combination with NaCl. In general, growth kinetic parameters were more affected by combining salt and PCS than by PCS only. Leroi et al. [77] stated no synergistic effect between salt and phenols, whereas LAB was more affected by salt than phenol content. Moreover, in the present study, pH reduction following bacterial growth was not significantly influenced by PCS alone, while changes were observed for the PCS-salt combination. On the contrary, Valø et al. [89] showed a significant lower aerobic plate count (APC) of PCS-treated salmon due to reduction in pH and a_w_, combined with a higher TPC than in traditionally smoked salmon. Thus, the importance of combining barriers in the seafood system must be emphasised. Conditions present in a food environment as well as the effect of the smoking-process method used, could affect the growth and antimicrobial activity of LAB. Moreover, Racioppo et al. [90] used the Food Spoilage and Safety Predictor (FSSP) to model the effect of temperature, salt, liquid smoke, CO_2_, and nitrites on the growth of LAB in fermented smoked fish products, demonstrating that liquid smoke, followed by temperature and salt had the strongest effect on the fermentation process. Uyttendaele et al. [72] reported growth limitation of *L. monocytogenes* in smoked fish when combining low pH (5.5–6), and low a_w_ (0.93–0.94). The study of Aymerich et al. [65] also showed the importance of the food matrix, where the *Carnobacterium* strain displayed *anti*-listerial activity *in vitro* while the same activity was exhibited only in CSS with higher fat, lower phenol and higher acetic acid concentration. Moreover, real products are more complex than the broth system tested, and further investigation is needed in order to apply these strains to smoked products, such as CSS.

### 3.4 Experiment 4: Growth kinetics of LAB in fresh VP salmon fillets

Two LAB strains, *Leuconostoc* (Le.m.68) and *Carnobacterium* (C.d.468) were selected for inoculation of *pre-rigor* salmon filet, vacuum packed and stored at 4 °C. The strain selection was based on the ability to grow relatively uninhibited at 4 °C and expressed antimicrobial activity against *L. innocua* in the salmon juice inhibitory assay.

**Fig. 4:**
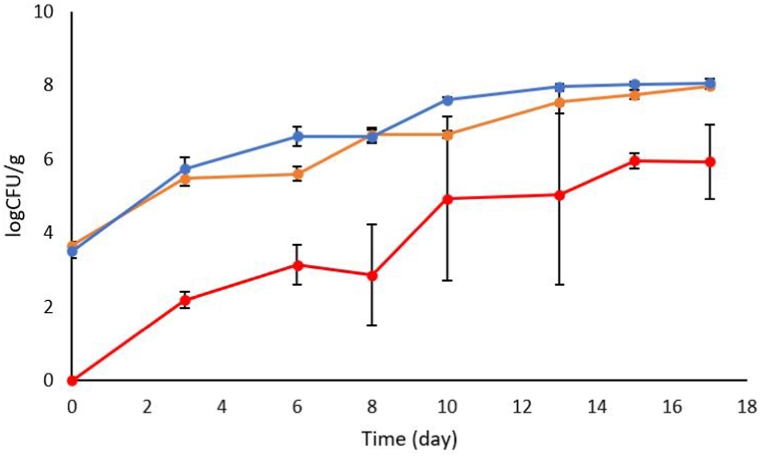
Growth of lactic acid bacteria in vacuum-packed salmon fillets stored at 4 °C. Inoculated with Le. m.68 ; Inoculated with C.d.468, and un-inoculated (control) . LAB growth was detected on MRS (Le.m68, control) or M17 (C d.468). The values are presented as log CFU/g ± SD (n = 3).

The strains were inoculated separately, and both strains were able to grow without any detectable lag phase in vacuum-packed (VP) salmon fillets, and their concentration increased by 4 log CFU/ml during the 17 d of storage (Table 8, Fig. 4). The inoculums were cold adapted for 2.5 d at 8 °C before the experiment, which can explain the lack of lag phases observed. Thus, a cold-adaptation step for the starter culture must be considered for an industrial application. LAB were also detected in the non-inoculated control sample, at a significantly lower initial level (p < 0.001), and their growth rate was comparable to the rate of the inoculated strains indicating that the inoculated strains were well adapted to the conditions in the salmon. No significant differences in the growth of the two LAB strains at the media used (MRS and Lyngby's Iron agar) indicate that the inoculated LAB strains dominated the fish microbiota, while differences were observed for the uninoculated salmon sample (p < 0.001).

**Table 8:** Growth kinetic parameters (lag phase (h), maximum growth rate, *μ*_*max*_ (1/h), final concentration Y_max_ (log CFU/g) for the selected LAB and LAB quantified on MRS/M17 in the uninoculated control (n = 3), during storage for 17d at 4 °C. Small letters (^ab^) indicate significant differences between the groups (per coloumn), calculated by one-way ANOVA (Tukey HSD).

Strain	Initial value	μ_max_	Y_max_	R^2^	SE	Lag phase
	logCFU/g	1/day	logCFU/g			day
Le.m.68	4.0386 ± 0.292^a^	0.292 ± 0.0441^a^	7.91 ± 0.313	0.937	0.363	NL
C.d.468	3.953 ± 0.356^a^	0.395 ± 0.0655^a^	8.019 ± 0.249	0.929	0.418	NL
Control	0.409 ± 0.449^b^	0.403 ± 0.0652^a^	6.0072 ± 0.56	0.926	0.566	NL
p-value	p < 0.001	p = 0.080				p = 0.263

No observed colour changes nor off odours were noticed by subjective observation of inoculated samples compared to control samples during every sampling. However, the spoilage potential of these strains will be elucidated in further studies. A prerequisite for applying any LAB strain in biopreservation is the lack of product spoilage due to the metabolic activity of the inoculated strain.

Several studies have reported a connection between *Carnobacteria* and the spoilage of seafood products [13,91,92]. In the study of Schirmer et al. [93], *C. maltaromaticum* was linked to the spoilage of fresh salmon. Quality deterioration of food products is also frequently related to *Leuconostoc* spp. since species from this genus can cause slime formation, gas production, and unpleasant odours [36]. However, interspecies and intraspecies variation in metabolic pathways and products are reported [29]. The presented results in the present study show that the tested strains are promising candidates for use in biopreservation of fresh and processed salmon products. Given the high antimicrobial activity and ability to grow under various growth conditions, further exploration of spoilage potential, antimicrobial activity against pathogens in a real product and the effect on the product's microbial community is required before applying the strains in industrial biopreservation.

## 4 Conclusion

To select LAB strains for biopreservative purposes of fresh and processed salmon products, they must fulfil some requirements, such as antimicrobial activity and growth in low-temperature storage conditions. The growth properties and modes of action (competition and/or production of antimicrobial compounds) are essential for food application. Two of the ten LAB strains in the present study showed bacteriocin-like activity, while the rest performed antimicrobial activities probably due to nutrient competition. All strains were able to grow at lowered temperatures in salmon juice and under different process parameters (salt, liquid smoke, and a combination of those) in BHI broth. The ability to grow under presented conditions is more likely species-dependent where origin, psychrotrophic nature and stress response play an essential role. Two selected strains were able to grow in VP salmon at 4 °C for 17 d storage period. Overall, results from this study demonstrate the potential of selected LAB for use in the biopreservation of fresh and processed salmon products. Thus, further investigation is fundamental for selecting the tested strains without the ability to cause spoilage and physicochemical deterioration of the desired salmon product.

## Author contribution statement

Jelena Stupar: Conceived and designed the experiments; Performed the experiments; Analyzed and interpreted the data; Wrote the paper.

Sunniva Hoel: Conceived and designed the experiments; Analyzed and interpreted the data; Wrote the paper.

Sigrid Strømseth: Performed the experiments; Analyzed and interpreted the data.

Jørgen Lerfall, Turid Rustad: Conceived and designed the experiments; Analyzed and interpreted the data.

Anita Nordeng Jakobsen: Conceived and designed the experiments; Analyzed and interpreted the data; Contributed reagents, materials, analysis tools or data; Wrote the paper.

## Data availability statement

Data will be made available on request.

## Declaration of competing interest

The authors declare that they have no known competing financial interests or personal relationships that could have appeared to influence the work reported in this paper.
